# Lunacy and Lunatic Asylums of Ireland
*The Sixth General Report on the District Criminal and Private Lunatic Asylums in Ireland. With Appendices. Presented to both Houses of Parliament, by Command of Her Majesty. Dublin. Printed by Alexander Thom, 87, Abbey Street, for Her Majesty's Stationery Office. 1853.


**Published:** 1853-10-01

**Authors:** 


### Art. IV.—LUNACY AND LUNATIC ASYLUMS OF IRELAND.*

We have repeatedly had our attention called to the deplorable state of lunacy in Ireland. Perhaps no land so highly favoured by Nature was ever so preyed upon by disastrous causes from without, exciting an intelligent and active minded-people to dissatisfaction, agitation, insurrection, self-expatriation,—and shall we not add insanity? Amidst the fierce moral tempest which has here raged with fluctuating intensity, driving a naturally excitable race to the opposite extremes of prostrate mendicancy and wild enthusiasm, we might well expect to find crime and insanity going hand in hand from the workhouse to the gaol, and from the overcrowded gaol to such lunatic asylums as might be open for their reception. "Alas! poor country, almost afraid to know itself!" faction-torn, fever-stricken, emigration-abandoned—we may truly image to ourselves the poor Irish peasantry in a state of unthinking and frantic desperation, resembling the terror-stricken victims on the precipitous and perilous rocks in Martin's picture of the Deluge—men, women, and children together mingled—throwing their arms wildly about them, not knowing whether in resistance or despair. The healthy springs of all human action paralyzed and radically contaminated, what else could be anticipated, but that disease and misery would do their worst? Yet how inadequate has ever been the provision made for this destitute multitude, upon whom humanity herself seems to have turned her back, leaving them no place of refuge, not even asylums, using that word in its proper sense, for the reception of the insane. The Report which we have just received, the sixth upon the District Criminal and Private Lunatic Asylums of Ireland, would, however, appear to indicate that government has in some degree thrown off its lethargy, and is bestirring itself; but the evidence before us is still discouraging, inasmuch as it holds out no prospect of any measure sufficiently comprehensive being adopted for the relief of a large class of sufferers, who have the strongest claims upon our sympathies. We do not wish to surcharge or over colour, in the least degree, the misery to which we have so often referred,† and would

* The Sixth General Report on the District Criminal and Private Lunatic Asylums in Ireland. With Appendices. Presented to both Houses of Parliament, by Command of Her Majesty. Dublin. Printed by Alexander Thom, 87, Abbey Street, for Her Majesty's Stationery Office. 1853.

† See the *Psychological Journal*, vol. ii., pp. 390, 391.

willingly avail ourselves of any authentic evidence which presented signs of progressive amelioration.

In taking up any report upon the state of lunacy in Ireland, the first question which suggests itself is, whether adequate accommodation exists for the reception of its insane poor? And for many years past we have been bound to answer this interrogatory with an emphatic negative. We have first to consider the extent of insanity in Ireland; how far the disease prevails; the numerical proportion of the population which may be so afflicted—and here, we regret to say, upon the very threshold of the inquiry, the inspectors leave us in the dark, and make a very lame apology for not supplying us with this very important statistical information. They set out with stating that as in their previous reports, they have entered into minute statistical details; they purpose on the present occasion, "whilst affording a general outline of the state of this department of the public service, confining themselves more particularly to the alterations and improvements which have been effected in it within the last two years." They therefore abstain from giving us the usual returns, showing the number of lunatics confined in private asylums, in poor-houses, and gaols, and take no notice of the wandering idiots, imbeciles, and lunatics, the number of which was, in 1846, returned as 6217; in 1849, as 6000; and, in 1851, as amounting to as many as 8985. Again, in the year 1846, there were 1921 lunatics confined in the poor-houses; in 1849, there were 1940; and, in 1850, the number was increased 2393. The present Report gives no statistical return whatever of either of these classes; and we should like, therefore, to know how, without this information, we can form any opinion of the adequacy of the provision, which is described in the Report before us, to accommodate the pauper lunatics of Ireland? This much appears obvious, that, in the year 1846, there were 8138 lunatics; in the year 1849, there were 7940; and in 1850, there were 11,378 returned as domiciled in poor-houses and wandering about the country. We therefore cannot understand how it happens that the inspectors, in the present Report, express themselves not only in terms of self-complacency, but in a tone bordering upon exultation upon having *four* new asylums (Cork, Kilkenny, Dublin, and Killarney), open, and three (Mullingar, Armagh, and Sligo,) nearly ready for the reception of patients, which, together with all the other asylums in the county, will not afford accommodation even for 5000 patients.

"The full accommodation" (we quote the words of the Report) "between existing public institutions and those in progress for the destitute insane of Ireland, may, within another twelve months be set down as adequate for 4500 patients. Taking the statistical ratio of lunacy in the general population, this amount of accommodation is by no means *too great*."

"Too great," indeed! Why we have just seen that, in 1850, there were as many as 11,378 lunatics eligible for admission; and the inspectors themselves avow that they believe insanity to be on the increase. We repeat, therefore, that the tone they assume appears to us unintelligible.

"Experience," they observe (we again quote the report), "fully justifies us in stating, that while *we have as accurately as possible apportioned the supply to the want* (!) it is far more beneficial for the community at large to have an excess of accommodation than the reverse, as the statistics of insanity furnish incontestable proofs that mental derangement is, unfortunately, on the increase, and that the only certain and legitimate means of arresting its progress is to be prepared to meet it; as proof of which, allusion need but be made to the recognized fact that the curability of the disease is mainly dependent on the facilities of an early treatment."

With this reasoning we are perfectly satisfied; the fact referred to is indisputable; but where is the supply to be found which will be "*apportioned*" to the wants of the 11,378 lunatics, whose numbers must, upon the evidence of the inspectors themselves, during the succeeding three years, have considerably augmented? We have not a shadow of evidence in the report before us that any supply at all adequate to the accommodation of the lunatic poor of Ireland has been provided; but the contrary appears too palpable upon the surface of the Report, for a provision for only 4500 will not meet even half of the declared exigency. This will appear more manifest from the following very imperfect table, which we have drawn up from the reports before us, showing what was lately acknowledged to be the extent of lunacy in Ireland.

*Table showing the Extent of Lunacy in Ireland.*

| | 1846 | 1849 | 1851 | 1852 | 1853 |
|---|---|---|---|---|---|
| In District Asylums . . | 2555 | 2603 | 2913 | 2722 | 2870 |
| In Local Asylums . . . | 562 | 365 | Included in District A. | No return | No return |
| In Gaols . . . . . . . | 290 | 338 | 280 | No return | No return |
| In Poor-houses . . . . | 1921 | 1940 | 2393 | No return | No return |
| Wandering and Unprovided | 6217 | 6000 | 8985 | No return | No return |
| In private Asylums . . | 251 | 432 | 456 | No return | 423 |
| Under the care of the Court of Chancery, but not in Asylums . . . . . | 76 | — | — | — | — |
| Criminal . . . . . . . | — | — | 91 | — | — |
| Total . . . | 11,872 | 11,678 | 15,118 | — | — |

Incomplete as this table necessarily is, it affords conclusive evidence that the extent of lunacy in Ireland far surpasses the contemplation of the inspectors, or at least the provision which they seem to conceive adequate to adjust the balance between the demand for the accommodation of pauper lunatics and the supply provided for them. We greatly fear—indeed it is evident—that the scale must greatly preponderate on the side of the demand; and the philosophy of being "prepared to meet" and "arrest" the progress of the disease "melts into air—thin air"—the very attenuated air of an Irish atmosphere!

But to proceed. Our attention is next called, in the present Report, to a curious fact, nevertheless one which we might have anticipated; it is simply this, that the sane and the insane in Ireland have a great horror of Irish lunatic asylums; and hence, when one of these noble edifices, upon which the inspectors congratulate themselves, is finally erected and thrown open, it fills very slowly. Little more than a century ago, Swift, from the context of his will, appears to have entertained some doubt whether fifty "madmen" could be found in all Ireland; and we are informed that years elapsed before some of the new district asylums were filled. For example, the Maryboro', built for 104 patients, in a district containing above 500,000 inhabitants, did not receive its full complement for three years; but what then followed? In the fifth it "became crowded beyond its original intent, whilst many cases were denied admission." Here we come at once to the root of an evil, which we have no doubt prevails to a prodigious extent in Ireland; —nay, we are informed that "similar observations might be made with reference to other institutions, which in time became obviously too restricted for the pressure on them; while the only mode of meeting the emergency was by having recourse to temporary additions, and the conversion of day-rooms and workshops into dormitories; also by taking of fresh land to afford out-door employment to the patients, who in some instances nearly doubled their primary numbers;" a state of things which, it is added, seriously interfered with a regular system of classification, and greatly augmented the number of the insane confined in gaols and workhouses. Can we be surprised at this? We have first an inadequate amount of accommodation, which, notwithstanding the erection of additional asylums, is, we maintain, upon the evidence before us, still the case; and, secondly, it does not appear that the parish authorities make any effort to bring the wandering idiots, imbeciles, and lunatics, from their forest haunts, where, in "*looped and windowed raggedness*," they are doubtless exposed, and perhaps inured, to all inclemencies of weather. In England, where every effort is made to suppress vagrancy, it is compulsory upon the part of the parish authorities,—overseers, constables, and relieving officers,—to apprehend all wandering lunatics, with the view of their being placed under proper care and control; but this is obviously not the case in Ireland, where, as is manifest from the table before us, large numbers are permitted to roam at large. Nor do the lunacy inspectors themselves appear even to calculate upon making adequate provision for them. Assuredly this is to be lamented. Although the asylums to which they refer filled slowly, it is admitted that they became gradually so overcrowded that it was found necessary to relieve them by finding temporary accommodation for the insane in places not adapted to the purpose; hence the circumstance of these asylums having filled tardily should afford no argument for the future provision being narrowed. The very contrary is the inference which ought to be deduced. To relieve gaols and workhouses, and to provide for the lunatic poor wandering at large, a more liberal and comprehensive measure than that which is described in this Report must be adopted. We are, however, to be thankful, we suppose, for the boon which has already been granted; and it certainly is a satisfaction to know that the new asylums which have been erected, and which are in progress, will in some measure increase the accommodation. It is well observed in the report that "the benefits derived from these establishments, not only in a curative point of view, but in their social and moral tendency, largely counterbalance the public expenditure which they entail." The inspectors furthermore add—and the observation seems to fasten upon them the necessity of adopting a more enlarged policy—that if ample provision for the treatment of insanity had in the first instance been made, "it would have had the effect of essentially obviating the propagation of a disease so prone to diffusion, and would not only have prevented an increased subsequent cost, but many unfortunate occurrences might have been guarded against." This acknowledgment pleads conclusively in favour of the views we have adopted; but the expenditure, in great as well as in little matters, is always the sore point, and our philanthropy we fear would press heavily upon the Irish exchequer. From the tabular synopsis which is here given, we find that the aggregate cost of the eight new district asylums amounted to 209,463*l.* 6*s.* 2¼*d.*, and the return to the Treasury of the sums assessed upon the several counties to meet this expenditure is given in detail; but into these financial and fiscal arrangements we forbear entering. The interest attached to them is purely local; but the protection provided for the lunatic, who is in reality to be regarded as a "State care," concerns humanity at large. It is an old axiom in English law that the reigning sovereign, in the capacity of *parens patriæ*, should, in return for the allegiance of the subject, take care of the person and property of those who are from insanity incapable of governing and defending themselves; hence the Lord Chancellor, as keeper of the great seal, is delegated by royal warrant to preside as the representative of the crown over this department of the public service; and in England it has been wisely provided by different and successive acts of parliament, that every county shall make adequate provision for its own pauper lunatics. The progress of legislation in Ireland, since the Union, has unhappily been retarded by the political events which have notoriously darkened its horizon; but a brighter day has now dawned, and her public lunatic asylums will, we trust, be put upon the same footing as those upon this side of the Channel. It was in the reign of George IV. that district asylums for the reception of pauper lunatics began to be erected; and, without entering into their history, which will be found in a previous number of this Journal,* we may briefly state that there are now thirteen of these district asylums, and the total number of the insane remaining in them collectively, on the 31st March, 1853, amounted to 2870. "Fully one half of these patients (we are informed by the Report) may be considered as incurable, and hence a question arises whether a large proportion of them might not be removed to union workhouses and other receptacles, where they might be supported at a diminished expense." This, we are of opinion, would not be a step in the right direction. The insane ought not, for the sake of the sane, to be transferred into such establishments—incurable, harmless, and tranquil as they may appear, they would require separate wards, and separate treatment. The inspectors truly add, that "the larger number of lunatics, the chronic inmates of asylums, although quiet and amenable under that supervision which asylums are peculiarly calculated to afford, would assume a very different character, and become dangerous if less systematically attended to." Experience sufficiently proves this to be the fact. The tranquil behaviour of a lunatic in an asylum is no criterion of what his conduct may be when released from a supervision of which he is habitually conscious, and which silently imposes upon him the necessity of self-restraint. A lunatic asylum, if it is to be conducted properly, must be an establishment *per se;* and the notion of conjoining it either with a gaol or a workhouse ought never for a moment to be entertained. Innumerable arguments—and those of the most cogent description—might be urged against such a proposition. It is unnecessary, however, at present to enter upon this discussion, as the inspectors state that they do not feel in a position at present to make any definite recommendation, and propose, therefore, instituting further inquiries on the subject. The result, we anticipate, will be fatal to a project which no pecuniary theory of economy can justify;—the wing of an asylum stretching out from the body of a gaol, or

* Vol. v., p. 526.

abutting from the side of an Elizabethan workhouse (the fashionable architecture at present of pauper buildings), would certainly have an anomalous aspect!

It is gratifying to observe that the sanitary state of the district asylums in Ireland has during the past year been favourable; and we are glad to recognise a tone of liberality in the Report respecting the dietary and domestic arrangements, the expediency of which experience has amply confirmed. We have much satisfaction in transcribing the following very judicious remarks:—

"As disease of the mind is so frequently found to be associated with physical debility, and not unfrequently arising therefrom, the importance of a well-regulated and generous diet needs no comment. We are happy to state that ameliorations in this respect are being effected. At the Richmond or Metropolitan Asylum, on a report from the physicians, animal food has been allowed either in a solid form with vegetables, or made into a nutritious soup, six times a week; whilst in other establishments, if not as liberally supplied, improvements in the general dietary have been made. The various boards of governors—though perhaps in some instances not to the extent we could wish—are still anxious to ameliorate, in regard to domestic comforts, the condition of the insane poor, and to act consistently with the public trust committed to them in a liberal manner towards the various officers and servants belonging to their respective asylums: hence, within the last two years in many instances, when we consider the scale of salaries and wages to be disproportionate to the duties entailed on the recipients, an increase of pay has been willingly accorded."

This really is only just; "the labourer is worthy of his hire." There is no position in life, no description of servitude, attended with so many anxieties and responsibilities as devolve upon officers of all grades who are resident in lunatic asylums; and, speaking generally, we have no hesitation in saying they are very much underpaid. The inspectors have favoured us in their appendix with the following table, which, compared with the one we have already given showing the amount of salaries given to the officers belonging to the county asylums of England, will be interesting. Being official, it is more complete than the one we were at the pains of drawing up, inasmuch as it presents us with the names of the respective office-bearers:—

*Return, showing the Names and Salaries of the principal Officers of District Lunatic Asylums in Ireland*, 31*st March*, 1853.

| ASYLUMS. | VISITING PHYSICIANS. | RESIDENT PHYSICIAN AND MANAGERS. | MATRONS. | PROTESTANT CHAPLAINS. | ROMAN CATHOLIC. | APOTHECARIES. | CLERKS AND STORE-KEEPERS. |
|---|---|---|---|---|---|---|---|
| | £ | £ | £ | £ | £ | £ | £ s. d. |
| Armagh . . | Thos. Cumming, M.D. 100 | Thomas Jackson . 200 | Matil. Jackson 50 | Not yet appointed . — | Not yet appointed.— | Vacant . . . . — | Samuel Parks . 30 0 0 |
| Ballinasloe . | Fred. Thornton, M.D. . 125 | John M'Kiernan . 200 | M. A. Callan . 60 | Rev. J. C. Walker . 40 | Rev. L. Dillon . 40 | J. E. Poyntz . . 30 | J. E. Maher . 55 0 0 |
| Belfast . . | Hy. M'Cormac, M.D. . 100 | R. Stewart, M.D. . 275 | M. F. Stewart 75 | Rev. John Carroll . 50<br>Rev. Wm. M'Cullagh, Pres. . . 50 | Rev. Patrick Fagan . . 50 | J. S. Mulholland 50 | Robert Lamont 50 0 0 |
| Carlow . . | Thos. O'Meara, M.D. . 100 | M. E. White, M.D. 260 | L. Parsons . .100 | Rev. Fred. F. Trench 25 | Rev. D. M'Carthy . . . 25 | H. Montgomery 25 | Timothy Brenan 40 0 0 |
| Clonmel . . | Wm. Jas. Shiell, M.D. 100 | James Flynn, M.D. 260 | Ellen Crofton . 73 | Rev. W. Palliser . . 30 | Rev. P. O'Neill . 35 | R. Grattan . . 30 | G. O'Neill . . 55 0 0 |
| Cork . . . | S. Hobart, M.D. . . 100 | Thos. Power, M.D. 430 | M. Merrick . 60<br>M. Smith, assistant m. . 50 | Rev. C. W. Clifford 50 | Rev. M. O'Sullivan . . . 50 | W. J. Jones . . 25 | W. Rennick, storekeeper 54 12 0<br>W. Connell, clerk . . . 25 0 0 |
| Killarney . . | W. W. Murphy, M.D. 100 | M. S. Lawlor, M.D. 260 | M. A. Falvey . 75 | Rev. G. S. Green . . 25 | Rev. D. Cotter . 50 | R. Linnegar . . 30 | J. Wallace . . 50 0 0 |
| Kilkenny . . | L. E. Kinchela, M.D. . 100 | Jos. Laylor, M.D. 260 | Joanna Ryan . 75 | Rev. J. Graves . . 30 | Rev. N. Kealy . 35 | J. Fitzsimons . 35 | W. O. Flahertie. 50 0 0 |
| Limerick . . | D. O'Callaghan, M.D. . 150 | R. Fitzgerald, M.D. 260 | M. A. Sleeman 70 | Rev. B. Jacob . . . 50 | Rev. L. Bunton . 50 | J. C. Bouchier . 30 | J. Bodkin . . 60 0 0 |
| Londonderry | F. Rogan, M.D . . . 100 | D. Cluff . . . 200 | E. Cluff . . . 50 | Rev. W. Wilson . 25<br>Rev. Dr. Denham, Pres. . . . . 25 | Rev. H. Nugent 25 | C. Morton . . 30 | R. Hamerton . 30 0 0 |
| Maryborough | John Jacob, M.D. . . 100 | T. C. Burton, M.D. 250 | E. Abbott . . 85 | Rev. T. Harpur . . 25 | Rev. N. O'Connor . . . 25 | T. Pilsworth . . 30 | J. Vanston . . 40 0 0 |
| Richmond . | J. Mollan, M.D. £168 9 4<br>R. Tuohill, M.D. 168 9 4<br>J. Banks, M.D., physician extraordinary, acting without salary<br>John Hughes, Surg. . 100 | Samuel Wrigley 250 | C. Wrigley . 80 | Rev. A. Leper . . 50 | Rev. J. Falkner . 50 | P. Beatty £27 13 8 | Clerk and storekeeper, vac, 50 0 0<br>Accountant, vacant . . 55 7 8 |
| Waterford . | W. Conolly, M.D. . . 100 | John Dobbs . . 200 | K. Ronayne . 70 | Rev. R. Bell . . . 25 | Rev. P. Wall . 25 | J. Mackesy . . 30 | T. Keary . . . 50 0 0 |
| Omagh . . | Hy. Thompson, M.D. . 100 | J. F. West, M.D. . 260 | H. Hudson . 75 | Rev. Richard M. Smith . . . . 30<br>Rev. Jos. Mitchell, Pres. . . . . 30 | Rev. D. O'Dogherty . . 30 | Francis Traynor 35 | John Carson . 50 0 0 |
| Sligo . . . | Wm. Little, M.D. . . 100 | J. M'Munn, M.D. 260 | Margt. Benson 75 | Rev. Edward Day . 30 | Not yet appointed. | John Lougheed . 35 | William Savage 50 0 0 |
| Mullingar . | Jos. Ferguson, M.D. . 100 | H. Berkeley, M.D. 260 | Deborah Long 75 | Not yet appointed . — | Not yet appointed. | Wm. Middleton . 35 | Benj. Barter . 50 0 0 |

By this Return it will be seen that to each of these asylums a visiting or consulting physician is attached, with a fixed, although nominal, salary, inasmuch as the amount of each salary is not commensurate with the *status* of the physicians, who doubtless regard the appointment in an honorary light; and we are further happy to observe that the inspectors enforce the propriety and expediency of these appointments by the following very judicious remarks. "Connected with every asylum"—we here again quote the Report—"is a consulting or visiting physician, whose services, in addition to those of a medical superintendent, may, by many, be deemed uncalled for; but affections of the mind are so complicated, and the consequences arising from them often so dangerous and unforeseen, that, though attended with expense, it is a judicious outlay—for if on any subject there obtains a greater variety of opinion, it is that on the existence of lunacy in certain parties; conclusions the most adverse being frequently arrived at on the same case by educated and experienced practitioners, a circumstance almost unknown in regard to corporeal disease."[1] We may add that this difficulty, this difference of opinion, is not limited to the medico-legal question of the existence or non-existence of insanity, but that it constantly arises in the treatment—medically and morally—of the disease in all its different stages—during the stage of convalescence not less than during that of incubation. We have, however, already, in the preceding pages, so fully argued this question, that we shall not here re-open the discussion; suffice it to say, that we are much gratified in finding our views so ably advocated, and practically adopted by the inspectors of lunacy in Ireland.

While on the subject of officers and attendants, the Report suggests that a superannuation fund should be annexed to these institutions; the inspectors state that they take the opportunity of expressing their conviction that "it is necessary for the well working of these great national establishments that a retiring allowance should be secured to those who have long and efficiently discharged their duties to the public and to those entrusted to their care within the precincts of an asylum—a place of all others which unfits a person advanced in years for after employment. At present there is no superannuation fund whatever, and thus we are occasionally obliged to retain the ineffective services of individuals who, having no means of support, to fall back upon, it would be an injustice to supersede." The cogency of this reasoning cannot, we apprehend, be challenged. We cordially approve of the suggestion; and we believe that in the large asylums in Paris the principle is adopted, and is found to work well. We are, indeed, at a loss as to what system should be adopted in order to secure the services of steady, humane, and intelligent attendants; it is, however, probable, that were some such prospective benefit and provision held out, it would encourage persons to remain attached for years to such asylums, instead of which the attendants and domestics are now constantly changing their places, which, apart from putting the proprietors to inconvenience, is a great disadvantage to the patients. A frequent change of nurses and attendants in asylums is a prodigious evil; the difficulty of finding eligible persons to hold these situations is universally acknowledged. A few years ago our own commissioners proposed annexing a *Bureau* for such applicants to their office in New-street, Spring-gardens, and addressed circulars to proprietors and superintendents of licensed houses requiring a return of the names and characters of discharged attendants; and a registry office, having a similar object in view, unconnected with the commissioners, has been opened in London, but the attempts to organize an improved system for ensuring the engagement of a better class of persons to fill these situations, have, we fear, hitherto been attended with little success. The root of the evil lies, we apprehend, to a considerable extent, in the fact that salaries and wages are, speaking generally, too small, and that there is no superannuation fund for those persons to look forward to, who may, in the faithful discharge of their duties, have grown grey in the public service. The suggestion, therefore, of the Inspectors of Lunacy for Ireland, merits serious consideration. Indeed, our impression is, that the difficulty referred to will be found an increasing one, now that the shores of Australia have thrown open golden prospects to the industrious among the working classes. And the fever of emigration is daily thinning our population. We would not willingly hazard a gloomy prophecy, but every day's experience will, we fear, prove the very great difficulty of finding persons willing, upon any terms, to become nurses, attendants, or servants in lunatic asylums.

The Report before us next re-opens the *quæstio vexata*, which was some years ago discussed and disposed of, respecting the admission of paying patients into the district asylums of Ireland. Here we are again at issue with the inspectors. They propose that pauper lunatic asylums should be open for the reception of private patients belonging to the middle classes of society—persons in trade, agriculturists, and an extensive class of the community who, on the one hand, cannot be placed in the category of paupers, and on the other, are devoid of sufficient means to meet the terms of well regulated licensed houses. We have elsewhere argued this subject; and can only repeat that the better classes of society not only object, very naturally, to being domiciled under the roof of paupers—but that the organization required for the management of a pauper lunatic asylum is, in all respects, so different, as to dietary, domestic arrangements, and general discipline, from that which we require in private asylums, that the two cannot well be combined. The evidence which was adduced upon the Committee of Inquiry presided over by Lord Monteagle, when the whole question of provision for lunatics in Ireland was entered into, was, we remember, clearly to the same effect. We cheerfully admit that every accommodation should be provided for the insane belonging to what are called the "middle classes of society"—but, at all events, upon this side of the Channel, there is no want of such establishments, for there are many licensed houses which receive patients at 12*s*., 15*s*., and 20*s*. per week; and we apprehend that persons in reduced circumstances who cannot afford to pay 12*s*. a week for lodging and maintenance come literally within the category of paupers. "On one point," says the Report, "we can pronounce with certainty, that in public establishments, the class in question would be more comfortably located than in private houses, inasmuch as in the former the stipend being small would go simply to the support of the lunatic without any derivable profit to a third party; moreover, large institutions could necessarily afford a greater amount of comfort and at much cheaper rate." Here the reasoning of the inspectors is utterly at fault; they are in error upon the *fact* to which they refer: it may be true that half a dozen patients at 12*s*. a-week would afford no profit to the asylum proprietor, but *numbers* may be made to pay even at this low figure. Indeed, we have no doubt that all the asylums in the metropolitan, and also in provincial districts, licensed for the reception of pauper as well as private patients, have derived considerable profit by conducting these establishments upon liberal principles. Be it also observed that the inspectors assign no reason whatever for the gratuitous assertion that patients would be afforded a greater amount of comfort in large institutions than in small asylums—but, on the contrary, they report officially their approbation of the manner in which these private asylums, conducted upon a small scale, are managed. "It is gratifying to us" (they state) "to be enabled to express ourselves favourably as to the humane and judicious manner in which these asylums are for the most part conducted." But this is not all. If the management of them be in the least defective, the inspectors who have the supervision of such establishments are themselves to blame. It is upon them the charge must recoil, for they are responsible that all asylums, large or small, under their jurisdiction shall be conducted upon proper, humane, and liberal principles. The total number of private patients confined in lunatic asylums in Ireland in 1853, amounted only to 423, of whom 245 were males, and 178 females. It is remarkable that in Ireland the proportion of male to female patients is greater in private than in public asylums, which the inspectors attribute to the circumstance that, in the more affluent grades of society, men having greater opportunities and being under less personal control than females, indulge more frequently in a course of life which leads to the development of insanity. With respect to the social condition of the inmates in private institutions, we find that in Ireland, as elsewhere, the numbers of the unmarried insane exceed the numbers of the married insane, the numbers being 258 unmarried, and 85 married.

| | Males. | Females. | Total. |
|---|---|---|---|
| Single . . | 199 | 139 | 338 |
| Married . | 46 | 39 | 85 |
| | 245 | 178 | 423 |

The most interesting portion of the present Report refers to the chaplaincy question—raised by the circumstance of the governors of the Belfast Asylum being adverse to the introduction of regular chaplains, and to the performance of public worship in that asylum; and having consequently refused admittance to three clergymen, a Protestant, Presbyterian, and Roman-catholic, appointed by government. The Belfast governors, in their memorial on the subject to the Lord-Lieutenant, state that they are in this dilemma—they have in the Asylum inmates belonging to *seven* different persuasions, and they require to know how *one* chaplain and *one* place of worship can satisfy the conscience of them all. The governors thus reason—"In an asylum" (we quote the memorial) "where there is but one chaplain, and one persuasion, the judicious clergyman may be permitted to visit without restriction; he may converse with them in their ward rooms, and have free intercourse with them collectively. How different would the case be here. There are in this house seven religious persuasions, three of them of considerable number. Are the different persuasions to be separated into different ward rooms on the visit of each chaplain? Are the chaplains to have free range of the house and grounds?" The difficulty is a curious one. Is one chapel to be dedicated to seven different religious persuasions? or is each sect to have a chapel of its own? How is one clergyman of the Established Church to perform service for the Catholic, the Presbyterian, the Trinitarian, the Unitarian, the Methodist, the Independent, and the Moravian? "The very fact," say the governors, "of three or four different kinds of religious services being performed each Sunday by three or four different clergymen under one roof, would, in a common sense view of the matter, be calculated to cause no small excitement even amongst the sane, to say nothing of that community being composed of insane individuals." What, then, is to be done? The inspectors answer, that it is not necessary to grant to every persuasion numerically in any institution a special clergyman, they therefore propose that a chapel in common shall be erected, where the chaplains may attend at different hours; and with respect to having a free passage through the house, they very truly remark, that "a judicious and sensible chaplain will, it is to be presumed, act with such good taste, discretion, religious quietude, and gentlemanly bearing, that his visits will, as at the asylums of Northampton, Surrey, Hanwell, Londonderry, Cork, &c., be always welcome;" but that "he will, of course, consult with the physicians as to the ministration of his office in detail." We remember a few years ago visiting, at Heidelberg, a church divided in the centre by a partition-wall, and were informed that on the one side the Lutherans performed divine worship, on the other side the Moravians; and certainly it struck us, *prima facie*, an anomaly that two separate altars for two separate religions should be raised under the same roof; but we are to consider that upon the great—the fundamental principles of the Christian religion, most sectarians are agreed, and their grounds of dissent do not remove any of them beyond the pale of Christianity, wherefore it is not as if we should propose to raise a Christian altar within the precincts of a Mohammedan mosque. We certainly see no objection to a chapel in common being attached to any institution where the inmates, though dissenting from each other in some opinions, are nevertheless agreed upon the great principles of the Christian faith. In lunatic asylums much discretion is certainly required in the administration of religious instruction and spiritual consolation; the patient's mental capacity to receive and understand what is said to him, and the effect which certain allusions may have upon excitable dispositions, must be well considered. The chaplain, however, if an intelligent man, assisted by the advice of the physician, will, in the exercise of his vocation, soon acquire sufficient tact and knowledge to direct and guide his ministrations. The late Dr. Millingen used to relate the story of a patient at Hanwell who always attended divine worship in the chapel of that asylum, and appeared very devotional. One Sunday, after he had paid great attention to the sermon, in which he seemed much interested, he was asked what he thought of it. "Oh," answered the man, "it was an excellent sermon, and all made so clear." "Well," rejoined the other, "but what was made clear? What was it all about?" "Why, you know *you* understood it," replied the lunatic; "it was all about the Emperor of Russia and the King of Prussia, to be sure!" We are not to conclude that because a congregation of lunatics are well conducted and attentive to the service, that they are therefore impressed with religious feelings and reflections. Many a silent and apparently devout listener is doubtless pondering upon his own delusions; but then, it may be argued, that if this happen with the insane, so does it often enough happen with those who are reputed sane, whose thoughts in church, particularly under the monotony of a stupid sermon, often wander far away from the devotional scene around them. But this is no argument against the performance of divine service in our churches and chapels on the Sabbath-day, neither ought it to be urged against the propriety of the insane being required to attend Sunday worship in the chapels annexed to public lunatic asylums. The effect of such service may, in some cases, be negative; but in others it undoubtedly is attended with positive good, having a soothing influence and curative tendency, by exciting the mind to healthy reflections. The inspectors have, on this subject, well remarked, that a great majority of the inmates of asylums are chronic cases—perfectly collected and rational except upon the immediate subject of their delusion. It therefore appears to them "a moral contradiction to encourage parties in the perusal of books of literature and amusement—botany, history, chemistry, *Chambers's Journal*, &c.—if they are to be denied, as incompetent to benefit thereby, the visitation of regular chaplains, and the privilege of attending divine worship in places set apart altogether for sacred purposes."

We cannot conclude this notice without referring to the success which has attended the establishment of the Central Asylum for the reception of what are termed "criminal lunatics," at Dundrum, in the vicinity of Dublin,* which has now for nearly three years been in full operation. Since the publication of their last Report, the inspectors inform us that material improvements have been effected in the general character and appearance of the institution. The land which, on the occupation of the premises not quite three years since, was in a poor and comparatively neglected condition, is now tastefully and judiciously arranged, and looks as if pertaining to a private residence. The portions immediately around the building are laid out in exercise and pleasure grounds; the garden affords an ample supply of vegetables for domestic use, and about ten acres are under oats and potatoes, the manual labour having been altogether performed by the patients. Besides the out-door labourers, there are some good tradesmen and mechanics among these "criminal lunatics" usefully engaged, and perhaps the best and most ingenious of them are those, we are told, who are occasionally the wildest in their delusions and the most dangerous. In the female department, both the insane and the convalescent, with few exceptions, industriously occupy themselves under the superintendence of the matron, and perform the common household

* Vol. v., p. 526, *loc. cit.*, where a full account of the early history of this asylum will be found.

duties of washing, spinning, knitting, mending clothes, &c. The healthy condition of the institution appears to have been unvaried; no epidemic or fatal disease is reported to have prevailed, and no accident from suicide or any incidental cause has taken place. It is observed that strangers, unless with permission from the proper authorities, are prohibited from inspecting the institution, which is obviously a very proper regulation, as many of the inmates, who may be in a convalescent state, naturally revolt at the idea of being made objects of public curiosity. Although the building is not protected by outer barriers, similar to those of many district asylums, and although the inmates have the liberty of exercising and employing themselves in the surrounding ground, which consists of twenty acres, no untoward occurrence has arisen, and no escape been effected, which is to be attributed to the vigilance and assiduous attention of the officers and attendants. "From the character and construction of the Central Asylum," the inspectors report, "the cost of the staff is considerably greater than in ordinary lunatic establishments. In the total absence of mechanical restraint or coercion of any kind, a full number of attendants is indispensable for the safe custody of the particular classes confined there." The total number at present in detention amounts to 109, of whom 69 are males, and 40 females; the following return exhibits the offences they committed, and their mental condition.

*General State of the Inmates in the Central Asylum, on 31st March*, 1853.

| Offences. | M. | F. | Total. | Mental Condition. | M. | F. | Total. |
|---|---|---|---|---|---|---|---|
| Homicide . . . . . . . | 32 | 20 | 52 | Recovered . . . . . . | 8 | 5 | 13 |
| Violent Assault . . . . | 21 | 6 | 27 | Improved. . . . . . . | 7 | 10 | 17 |
| Burglary, Arson, Felony . | 16 | 14 | 30 | Insane . . . . . . . . . | 54 | 25 | 79 |
| Total . . . . | 69 | 40 | 109 | Total . . . . | 69 | 40 | 109 |

By this return it will be observed, that of these 109 criminal lunatics, the offences of whom varied in degree from the perpetration of a misdemeanor of petty larceny to the commission of homicide or attempt to murder, 17 are reported to have improved, and 13 recovered; and a grave question here arises, how are these recovered persons to be disposed of? There are two points for consideration:—First. The inspectors state that at the present annual rate of admission, in the course of a few years, the Dundrum Asylum will be found too limited for the demand, unless it be relieved by full discharges, or the removal of patients who may become fatuous and decrepit to their respective district hospitals. Secondly. Under what circumstances ought a recovered patient to be liberated? It is evident that unless the Central Asylum be relieved by discharges or removals, the number of sane inmates who have recovered must accumulate, which cannot be otherwise than a great evil. The inspectors therefore state, that on mature consideration of the subject, they are disposed to think that "were it merely to serve by way of example and encouragement to good conduct, it would be advisable to occasionally liberate recovered patients, but not without a minute examination into the antecedents of each case, and with a scrupulous regard to the feelings and just prejudices of the public at large." Upon many other grounds this proposition of the inspectors may be defended, but we at present must abstain from entering upon this subject; and in closing the Report before us, we can only repeat, that although much has been done to provide additional accommodation for, and ameliorate the state of, the insane poor of Ireland, for which a large debt of gratitude is due by the country to the inspectors, Messrs. White and Nugent, much still remains to be effected, which we have no doubt their zeal and perseverance will eventually accomplish. All public benefactors have great difficulties to surmount; and those which the inspectors of lunacy in Ireland still have to contend with, are of no ordinary description. May success, however, attend their continued philanthropic exertions!

---

